# The Impact of Nanoparticles on Proton Therapy: A Review Study

**DOI:** 10.3390/cancers18182904

**Published:** 2026-09-08

**Authors:** Georgios Nikiforos Ntoumas, Dimitra Desse, Anna Zygogianni, Andromachi Kougioumtzopoulou, Dimitrios Alexandros Angelakis, Georgios Patatoukas, Nikolaos Kollaros, Ioannis M. Koukourakis, Vasileios Kouloulias, Kalliopi Platoni

**Affiliations:** 1 Department of Clinical Radiation Oncology, Medical School, “Attikon” University Hospital, National and Kapodistrian University of Athens, 124 61 Athens, Greece; dimidesse@gmail.com (D.D.); azygogianni@med.uoa.gr (A.Z.); ankougio@med.uoa.gr (A.K.); alexandrosangelakis24@gmail.com (D.A.A.); ikoukourakis@med.uoa.gr (I.M.K.); 2 Department of Applied Medical Physics, Medical School, “Attikon” University Hospital, National and Kapodistrian University of Athens, 124 61 Athens, Greece; gpatatouk@med.uoa.gr (G.P.); nkollaros08@gmail.com (N.K.); vkouloul@med.uoa.gr (V.K.)

**Keywords:** proton therapy, nanoparticle, cancer cells

## Abstract

**Background/Objectives**: Proton therapy provides superior dose conformity and better normal tissue sparing than conventional radiotherapy but is limited by its relatively low relative biological effectiveness (RBE). Nanoparticles have emerged as promising proton radiosensitizers capable of enhancing physical dose deposition and biological responses. **Methods**: A narrative literature review was conducted using PubMed, Scopus, Web of Science, and Google Scholar. **Results**: Gold nanoparticles were the most extensively studied radiosensitizers, while platinum, gadolinium, hafnium oxide, titanium dioxide, iron oxide, and other nanoparticles also demonstrated promising radiosensitizing effects. Evidence indicates that nanoparticles enhance local dose deposition, increase LET and ROS production, promote DNA damage, and improve tumor control. **Conclusions**: Nanoparticle-assisted proton therapy represents a promising strategy for improving cancer treatment by increasing therapeutic efficacy while preserving healthy tissues. Although preclinical studies demonstrate significant improvements in therapeutic efficacy, challenges related to tumor targeting, biodistribution, safety, and clinical validation remain. Further research is required to facilitate clinical implementation.

## Simple Summary

Proton therapy provides superior dose conformity and better normal tissue sparing than conventional radiotherapy but is limited by its relatively low relative biological effectiveness (RBE). Nanoparticles have emerged as promising proton radiosensitizers capable of enhancing physical dose deposition and biological responses. This review summarizes current advances in nanoparticle-assisted proton therapy, highlighting metallic and metal oxide nanoparticles and their mechanisms of action, including increased secondary electron production, reactive oxygen species generation, and enhanced DNA damage. Although preclinical studies demonstrate significant improvements in therapeutic efficacy, challenges related to tumor targeting, biodistribution, safety, and clinical validation remain. Further research is required to facilitate clinical implementation.

## 1. Introduction

Radiotherapy is essential in cancer treatment, aiding more than 50% of diagnosed patients. Advanced radiotherapy treatments utilize X-rays or gamma rays to accurately target tumors, administering intense radiation doses directly to the affected region. Nonetheless, the collateral damage inflicted by these rays on healthy tissues adjacent to the tumor and along the radiation trajectory is substantial, resulting in side effects that can adversely affect the patient’s quality of life [1]. In contrast to X-rays, which gradually diminish in intensity as they penetrate matter upon entering the body, energetic protons or heavy ions primarily release the majority of their energy at the conclusion of their ranges, referred to as the Bragg peak [2].

The characteristic depth–dose profile of proton beams enables highly conformal dose delivery while reducing irradiation of surrounding normal tissues, thereby offering a favorable therapeutic ratio compared with conventional photon radiotherapy [3,4]. In clinical practice, a generic relative biological effectiveness (RBE) value of approximately 1.1 is conventionally applied; however, proton RBE is not constant and may vary according to LET, dose, biological endpoint, tissue type, and position within the proton beam [5]. The principal clinical advantage of proton therapy therefore lies in its favorable dose distribution rather than an intrinsically high RBE. In this context, nanoparticles have attracted increasing interest as radiosensitizers capable of further enhancing the physical and biological effects of proton irradiation [6].

The ability of nanoparticles to efficiently interact with biological tissues and to preferentially accumulate within tumors lies in their small dimensions, typically below 100 nm, the high surface-area-to-volume ratio, as well as the enhanced permeability and retention effect (EPR) following systemic or intratumoral administration [7]. The above properties, coupled with their favorable biocompatibility, support their use as radiosensitizers through the modification of radiation-induced energy deposition and biological responses, resulting in increased tumor-cell damage [8,9,10]. High-atomic-number (high Z) materials, including gold, platinum and gadolinium, have been particularly studied in the context of photon radiotherapy, due to their strong interactions with X-rays [11,12]. In proton therapy, however, nanoparticle-mediated enhancement of radiation effects may involve different interactions with matter, which may include increased production of secondary electrons and other secondary particles, together with physicochemical processes contributing to local energy deposition and radiosensitization [6].

A closer examination of these occurrences reveals that protons partially lose energy when traversing nanoparticles, whether composed of Low-Z or High-Z elements [13]. The protons, when exiting the nanoparticles, exhibit a reduced velocity compared to their entry, and this alteration in kinetic energy will influence both the Linear Energy Transfer (LET) and biological effectiveness. The literature indicates that protons (ions) primarily interact with orbital electrons and also with atomic nuclei, contributing to electronic and nuclear stopping power [14]. Consequently, protons will dissipate their kinetic energy by repeated scattering as they approach the endpoint. Incorporating High-Z nanoparticles into the tumor will result in protons dissipating their energy at an accelerated rate. Thus, the use of High-Z nanoparticles will enhance radio-sensitization by producing additional secondary electrons and decelerating protons, which have increased capacity for dose delivery due to their larger linear energy transfer (LET) [15]. The influence of AuNPs on proton energy deposition and LET has been investigated primarily through simulation studies. For example, Belousov et al. reported, using a simulated homogeneous mixture of water and gold, that the LET of protons increased in the presence of gold atoms [16].

Previous reviews have addressed nanoparticle-mediated radiosensitization in ion-beam therapy and proton therapy, including a recently published review focusing exclusively on nanoparticle-mediated enhancement of proton therapy [6]. However, important questions remain regarding the comparative suitability of different nanoparticle classes for proton irradiation, the distinction between proton-specific and photon-derived evidence, and the factors limiting clinical translation. The present review provides an updated and broader synthesis of the available evidence by considering metallic, metal oxide, and multifunctional nanoparticles and by integrating the physical, chemical, and biological mechanisms involved in proton radiosensitization. In addition, particular attention is given to emerging targeting strategies, combination approaches, and the translational challenges associated with biodistribution, safety, experimental standardization, and clinical implementation.

## 2. Materials and Methods

A narrative literature analysis was conducted to evaluate the role of nanoparticles in enhancing the biological and physical effectiveness of proton therapy. The literature search was performed in July 2026 across databases such as PubMed, Scopus, Web of Science, and Google Scholar, implementing the following combined terms related to proton therapy and nanoparticle-mediated radiosensitization: “proton therapy”, “proton irradiation”, “nanoparticles”, “radiosensitization”, “gold nanoparticles”, “high-Z nanoparticles”, “metal oxide nanoparticles”, “dose enhancement”, “linear energy transfer”, “relative biological effectiveness”, and “reactive oxygen species”. Boolean operators “AND” and “OR” were applied to generate the appropriate search strings for each database. Peer-reviewed original research articles, experimental studies, Monte Carlo simulation studies and relevant review articles published in English were considered for inclusion. No publication-date or geographical restrictions were applied. Particular attention was given to studies exploring the underlying mechanisms involved in nanoparticle and proton beam interactions, physical dose enhancement, secondary-particle production, radiochemical effects and biological outcomes. Studies investigating photon or other forms of ionizing radiation were also included, considering they provided mechanistic or translational information relevant to nanoparticle-mediated radiosensitization; however, evidence derived from these studies was distinguished from proton-specific findings where appropriate. Finally, reference lists of review articles were additionally searched to identify potentially eligible studies. Given the narrative nature of the review, no formal risk-of-bias or study-quality assessment was performed.

## 3. Results

**Figure 1:**
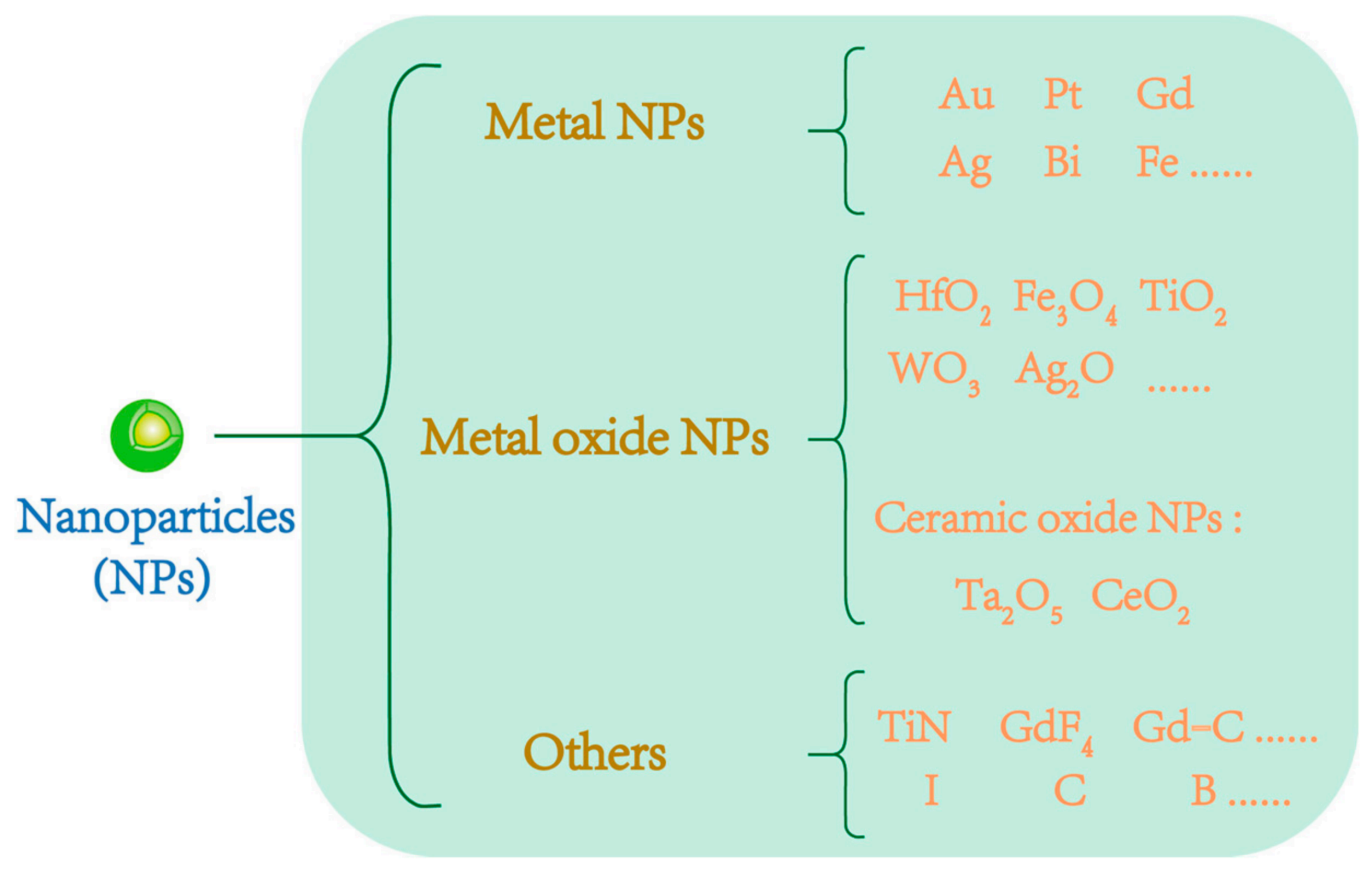
Classification of nanoparticles (NPs) with potential radiosensitization effects in proton therapy.

Various nanoparticles have been investigated for their possible radiosensitizing effects in proton therapy [17]. The nanoparticles that have been extensively studied and demonstrated specific advantages in experimental or simulation research can be classified into several categories, including metal nanoparticles, metal oxide nanoparticles—which encompass various ceramic oxides—and other nanoparticle forms [6] (Figure 1).

### 3.1. Metal Nanoparticles

Metal nanoparticles exhibit a pronounced radiosensitization impact in X-ray radiation, attributable to their elevated atomic number and modifiable surface chemical properties [18,19]. Numerous studies have shown that these nanoparticles can improve physical dose deposition, hence intensifying radiobiological effects in proton therapy [20].

#### 3.1.1. Gold Nanoparticles

Gold nanoparticles are acknowledged as among the most promising metallic radiosensitizing nanoparticles, despite their comparatively high expense [21,22]. Their beneficial attributes, including elevated electron density, straightforward synthesis, diverse sizes, customizable surface functionalization, low cytotoxicity, efficient tumor targeting, and chemical stability, make them exemplary candidates for application as radiosensitizers in proton treatment [23]. Liu et al. documented the sensitization impact of gold nanoparticles in proton therapy by preliminary in vitro studies [24].

Subsequently, Lin et al. built a Monte Carlo model to examine the radiosensitization effects of gold nanoparticles during proton irradiation, forecasting a substantial nanoscale dose augmentation [25]. Polf et al. established that the internalization of gold nanoparticles by cells led to a 15–20% enhancement in the effectiveness of proton irradiation in prostate cancer cells in vitro [26]. Kim et al. performed in vivo tests utilizing Balb-c mice injected with CT26 cancer cells to validate the tumor growth suppression effect augmented by gold nanoparticles [27]. Their findings demonstrate a significant decrease in tumor volume growth when gold nanoparticles were utilized alongside proton irradiation, in contrast to mice that underwent solely proton treatment [27]. Additionally, in their simulation study investigating mechanisms underlying AuNP-mediated enhancement during proton irradiation, Tran et al. reported increased dose deposition in the vicinity of spherical gold nanoparticles and elevated production of radiolytic species, with secondary electron generation contributing to the formation of cytotoxic radicals [28]. These findings have encouraged further experimental and modeling investigations to clarify both the magnitude of AuNP-mediated radiosensitization and the mechanisms responsible for this effect [29,30,31].

Recent contemporary computational approaches have combined physical dose calculations with prediction of biological response. Velten et al. employed the simulation tools TOPAS and Geant4-DNA to study AuNP-associated energy deposition at both macroscopic and microscopic levels and incorporated these results into a biological model predicting the survival of MDA-MB-231 breast cancer cells [32]. In accordance with the findings by Lin et al., they further supported an association between AuNPs, enhanced dose deposition and augmented biological damage with proton beam irradiation [27]. Rajabpour et al. investigated how different Geant4 physics models influence simulated energy deposition and radiochemical yields around proton-irradiated AuNPs, highlighting the importance of model selection when interpreting nanoscale simulations [33]. Ahn compared AuNP-mediated dose enhancement under proton, helium-ion, and carbon-ion irradiation [34]. At 1 nm from the nanoparticle surface, the calculated dose enhancement ratio (DER) was 4.89, 4.86, and 4.70 for proton, helium-ion, and carbon-ion irradiation, respectively [34].

Experimental studies have also demonstrated biological effects associated with AuNPs during proton irradiation. In CHO-K1 cells, Cunningham et al. observed significantly reduced cell survival following irradiation with 200 MeV protons when AuNPs were internalized by the cells [35]. In addition to effects on cell survival, radiochemical mechanisms have been investigated. Using a fluorescence-based approach, Zwiehoff et al. evaluated ROS production associated with AuNPs during proton irradiation, providing evidence that enhanced ROS generation may contribute to indirect radiation-induced cellular damage [36]. Furthermore, Johny et al. demonstrated that AuNP surface chemistry and specific surface area strongly influence ROS generation during proton irradiation and evaluated the applicability of these nanoparticles in human medulloblastoma cells [37].

#### 3.1.2. Platinum Nanoparticles

Platinum nanoparticles have demonstrated radiosensitizing properties, including enhancement of radiation-induced DNA damage [38]. These nanomaterials can induce immunogenic cell death, thereby triggering the immune system to recognise and kill malignant cells [39]. Porcel et al. investigated the radiosensitizing potential of platinum nanoparticles (PtNPs) under ion irradiation [38]. During carbon-ion exposure, the presence of PtNPs increased local energy deposition through enhanced secondary-electron production, which was associated with greater DNA damage [38]. Further experimental investigations demonstrated that PtNPs could also enhance molecular damage under different irradiation conditions, including photon and ion beams [40].

Evidence from conventional photon radiotherapy further corroborated the radiosensitizing properties of PtNPs. Using Geant4-DNA simulations, Batooei et al. investigated radiation-induced effects associated with high-Z nanoparticles under 6 MV X-ray irradiation and additionally modelled PtNP-mediated radiosensitization in gastric adenocarcinoma cells [41]. Although these findings were obtained using photon irradiation, they contributed to subsequent interest in evaluating PtNPs specifically under proton irradiation. In this context, Ganjeh et al. compared the dose enhancement factor (DEF) of several nanoparticle materials under proton irradiation and reported a greater calculated dose-enhancement effect for platinum compared with the other investigated nanoparticles [42].

Zwiehoff et al. confirmed the augmented radiosensitization effects of platinum nanoparticles during proton irradiation by assessing singlet oxygen generation. They noted substantial plasmid DNA cleavage at a clinically pertinent proton dose of 5 Gy, leveraging the synergistic boosting effects of platinum nanoparticles and clinically acceptable stabilising ligands [43].

#### 3.1.3. Gadolinium Nanoparticles

Apart from gold and platinum, gadolinium nanoparticles represent another class of high-Z materials that have attracted interest for their radiosensitizing effects [44]. The simulations performed by Wälzlein et al. demonstrated that gadolinium nanoparticles cause an increase in local dose under proton irradiation, albeit lower than that observed for gold and platinum nanoparticles [45]. At the molecular level, Schlathölter et al. reported increased occurrence of both DNA single-strand breaks (SSBs) and double-strand breaks (DSBs) in the presence of GdNPs, including the formation of complex nanoscale DNA lesions [46]. Rovira et al. subsequently used Geant4-DNA simulations to compare the radial energy distributions produced around gold and gadolinium nanoparticles during proton irradiation, further demonstrating nanoparticle-associated changes in nanoscale energy deposition [47].

A comparative investigation by Hosseini et al. evaluated gold-, gadolinium-, and iodine-based nanoparticles under proton irradiation [48]. Consist with the findings of the above studies, they demonstrated that gadolinium nanoparticles could increase local energy deposition and DNA damage, providing additional evidence for their potential role as proton radiosensitizers [48].

Beyond their potential as radiosensitizers, gadolinium-based nanoparticles have already been proven valuable as contrast agents in magnetic resonance imaging and neutron capture therapy [49]. In radiotherapy applications, gadolinium is frequently incorporated into functionalized nanoparticle formulations rather than being used in its elemental form. In particular, gadolinium-containing polysiloxane nanoparticles have demonstrated radiosensitizing properties and have progressed to clinical investigation in combination with radiotherapy [50].

#### 3.1.4. Other Metal Nanoparticles

Alongside gold, platinum, and gadolinium nanoparticles, numerous other metallic nanoparticles, such as silver, bismuth, and iron, demonstrate the capacity to augment the radiobiological effects of proton treatment [29,42,45]. The initial simulation investigation examining the radiation enhancement effects of diverse high-Z elements was performed by Wälzlein et al. using the Monte Carlo program TRAX [45]. This study revealed that silver nanoparticles exhibit a degree of radiosensitization in proton therapy, with a local dose enhancement level akin to that of gadolinium nanoparticles [45]. Ganjeh et al. formulated a cellular size model to assess the dose-enhancing effects of gold, platinum, silver, iodine, and tantalum oxide nanoparticles, concentrating on the modeling of low-energy proton generation. The findings demonstrate the subsequent hierarchy of enhancing efficiency: platinum > gold > silver > tantalum oxide > iodine [42]. Rashid et al. investigated the molecular impacts of gold, platinum, bismuth, iron, and other metallic nanoparticles following 150 MeV proton irradiation. Their findings demonstrate a notable radiosensitization effect linked to these nanoparticles, especially bismuth nanoparticles, which showed a sensitization enhancement ratio of 4.93 and led to the highest ROS generation among the investigated nanoparticles in HCT116 cells relative to the control group [29].

### 3.2. Metal Oxide Nanoparticles

Specific metal oxides have exhibited considerable radiation enhancement effects in radiation therapy, exceeding the effectiveness of their respective elemental metals. A multitude of in vitro and in vivo research, along with simulation studies, indicates that certain metal oxides have significant potential for dose increase in proton therapy [17,51].

#### 3.2.1. Hafnium Oxide Nanoparticles

The biocompatibility, physicochemical stability, and potential for functionalization and tumor-directed delivery of oxide nanoparticles (HfO_2_ NPs) have supported their investigation as potential radiosensitizers [52].

HfO_2_ nanoparticle-mediated radiosensitization was primarily studied in combination with conventional external-beam radiotherapy. Early clinical evaluation consisted of intratumoral administration of HfO_2_ nanoparticles followed by external-beam irradiation in patients with soft-tissue sarcoma, and subsequent clinical trials have investigated this approach in various tumor types, including soft-tissue sarcoma, head and neck cancer, and liver cancer [53,54,55]. It should be emphasized that these clinical studies were conducted with conventional radiotherapy and therefore should not be considered clinical evidence for HfO_2_-enhanced proton therapy. Therefore, proton-specific evidence remains predominantly preclinical. Gerken et al. compared the response of gold and several metal oxide nanoparticles under photon and proton irradiation and demonstrated that HfO_2_ nanoparticles retained radiosensitizing activity under proton exposure [51]. Their findings indicated that this effect could not be explained solely by physical dose enhancement and highlighted the contribution of physicochemical and catalytic processes [51]. Further experimental validation is therefore required to determine the potential of HfO_2_ nanoparticles specifically in proton therapy.

#### 3.2.2. Iron Oxide Nanoparticles

Iron oxide, a prevalent and economical inorganic metal oxide, is significant in illness therapy owing to its advantageous biocompatibility and the capacity for functionalization through specific ligands, such as MRI contrast agents and drug delivery systems [56,57]. Recent studies have broadened the applicability of iron oxides to encompass their use as agents in photothermal therapy, magnetocaloric therapy, and as diagnostic tools in immunotherapy [58].

Iron oxide nanoparticles have demonstrated radiosensitizing properties under photon irradiation, with reported effects involving enhanced energy deposition and increased production of radiolytic species [59,60]. Regarding radiosensitization under proton beam radiotherapy, Brero et al. evaluated magnetic iron oxide nanoparticles in BxPC3 pancreatic cancer cells using a combined treatment strategy involving proton irradiation and hyperthermia [61]. In vitro, this combination reduced cell survival and was associated with increased DNA double-strand breaks and reactive oxygen species production. These findings suggest a radiosensitizing effect of such nanoparticles in this setting, although the interpretation of the contribution of nanoparticles should also take into account the accompanying hyperthermia [61].

Iron oxide nanoparticles have additionally been explored for imaging and proton-range monitoring. Ibáñez-Moragues et al. developed zinc-doped iron oxide nanoparticles and evaluated their response to proton irradiation. The nanoparticles enhanced positron emission tomography (PET) and prompt gamma signals, indicating their potential use as signal-enhancing agents for monitoring proton-beam range during treatment [62].

#### 3.2.3. Titanium Oxide Nanoparticles

Titanium dioxide (TiO_2_) is a relatively low-Z metal oxide nanomaterial having a high surface-area-to-volume ratio and favorable biocompatibility [63]. Guerreiro et al. compared the radiosensitizing properties of 22 metal oxide nanoparticles under 6-MV X-ray irradiation and found that TiO_2_ increased radiation-induced superoxide production [64]. Proton-specific evidence was subsequently provided by Gerken et al., who observed significant ROS production and biological enhancement in the presence of TiO_2_ during proton irradiation, with the radiosensitizing effect reaching approximately 290% at the highest concentration investigated [51]. This response was greater than that observed for hafnium, tungsten, and silicon oxide nanoparticles under the experimental conditions examined. Proton interactions with titanium can also induce nuclear reactions, including Ti(p,x)V processes that generate positron-emitting vanadium isotopes [51]. However, the extent to which such nuclear interactions contribute to the observed biological enhancement remains uncertain. Overall, the mechanisms underlying TiO_2_-mediated proton radiosensitization have not yet been fully elucidated and require further investigation.

#### 3.2.4. Ceramic Oxide Nanoparticles

Ceramic oxide nanoparticles are nanostructured ceramic substances extensively utilized in materials science, microelectronics, optics, and various other domains [65]. Tantalum pentoxide (Ta_2_O_5_) is acknowledged as a non-toxic, high-Z nanomaterial that, when internalized by cells, creates clumps around the nucleus, suggesting a protective shell. This differential intracellular distribution may provide different dose enhancement effects upon radiation exposure [66]. Brown et al. were the pioneers in demonstrating the effectiveness of Ta_2_O_5_ nanoparticles in augmenting radiation doses in vitro, indicating a notable decrease in the survival rate of 9 L gliosarcoma cells post-radiotherapy upon internalization of these nanoparticles [67].

Engels et al. developed a Monte Carlo model based on experimental measurements to calculate energy deposition around Ta_2_O_5_ nanoparticles during irradiation [66]. Their analysis demonstrated localized physical dose enhancement and indicated that its magnitude depended on factors including nanoparticle aggregation, their spatial position relative to the beam, and the incident radiation energy [66]. Proton-specific simulations were subsequently performed by McKinnon et al. using Geant4 to investigate nanoscale energy deposition around ceramic oxide nanoparticles, including Ta_2_O_5_ and cerium dioxide (CeO_2_) [68]. The calculated local dose enhancement was approximately 16% for Ta_2_O_5_ and 14% for CeO_2_, demonstrating a modest nanoscale physical enhancement under proton irradiation [68].

#### 3.2.5. Other Metal Oxide Nanoparticles

Alongside the aforementioned metal oxide nanoparticles, other oxides, including tungsten oxide and silver oxide, have exhibited radiosensitization capabilities in radiation therapy. The research by Gerken et al. showed that tungsten oxide enhances physical dosage during radiation therapy and increases reactive oxygen species due to surface catalysis in both photon and proton therapies [51]. Under photon irradiation, Liu et al. demonstrated that silver nanoparticles enhanced radiation-induced apoptosis in glioma cells [69]. In vivo, the combination of silver nanoparticles and radiation also prolonged survival in glioma-bearing mice [69]. The principal nanoparticle systems investigated specifically in the context of proton therapy and their reported radiosensitizing effects are comparatively summarized in Table 1.

### 3.3. Proton Radiation and Nanoparticles

The engagement of keV X-ray beams with metallic nanoparticles primarily relies on the physical principles of the Compton and Photoelectric effects. A photon striking the nanoparticle is absorbed, either completely or through an interaction in which only part of its energy is transferred, resulting in the production of energetic secondary electrons [70]. More generally, ionizing radiation can transfer sufficient energy to remove electrons from atoms or molecules, resulting in ion formation. Charged particles, including electrons, protons, alpha particles, and heavier ions, are classified as directly ionizing because they interact with atomic electrons through Coulomb forces and deposit energy directly along their trajectories. In contrast, uncharged radiation, including photons and neutrons, is classified as indirectly ionizing because its energy is transferred to secondary charged particles, which subsequently produce ionization within the surrounding medium [71]. In the case of photons, interactions such as the photoelectric effect and Compton scattering can generate energetic secondary electrons, which are, subsequently, directly ionizing. The absorbed dose is enhanced by strong photoelectric absorption in high-Z nanoparticles [70].

Should the expelled electrons be substituted, the energy emitted by electrons descending from elevated orbits culminates in either Auger electrons or fluorescence photons. Auger electrons possess a markedly restricted range of influence, although they can induce a considerably elevated ionization density in a confined region. The Auger electrons impart their energy within the vicinity of the AuNP, resulting in pronounced inhomogeneous dosage patterns at the nanoscale. Consequently, the integration of radiation with AuNPs is anticipated to enhance radiosensitization [70].

Gold nanoparticles (AuNPs) have been successfully employed as potential radiosensitizers in studies; nevertheless, the majority of current experiments are confined to relatively low-energy kilovoltage X-rays. Limited research has been conducted utilizing high-energy particles to irradiate cells or tumors that include nanoparticles demonstrating radiosensitization akin to that of X-rays. The majority of research suggests or forecasts a diminished effect of dose escalation for therapeutic protons compared to low-energy X-rays [25,72].

Nonetheless, the practical application of kilovoltage X-rays is constrained by their low penetration depth within the patient. In this context, protons represent an attractive alternative for combining nanoparticle radiosensitization with the favorable depth–dose characteristics of particle therapy [35]. Verkhovtsev et al. demonstrated that metal nanoparticles (MNPs) subjected to ion treatment significantly increased the secondary electron yield relative to pure water, owing to the excitation of plasmons within the nanoparticle. Specifically, noble metal nanoparticles have demonstrated superior performance relative to other nanoparticles (e.g., gadolinium) [73].

Experimental studies have investigated the interaction of metallic nanoparticles with proton irradiation and its potential biological consequences [26,27,29]. Among the mechanisms initially proposed to contribute to these effects are particle-induced X-ray emission (PIXE) and particle-induced gamma-ray emission (PIGE), in which proton interactions with nanoparticle constituents can result in the emission of characteristic X-rays or gamma rays [74]. Kim et al. investigated these processes in metallic nanoparticle–proton interactions and proposed that the resulting secondary radiation could contribute to nanoparticle-mediated enhancement [74].

In early experimental investigations, Kim et al. evaluated superparamagnetic nanoparticles in combination with proton irradiation and reported increased cytotoxicity following exposure to a 45 MeV proton beam [75]. Subsequent experiments investigated AuNPs, ligand-coated AuNPs (L@AuNPs), and iron nanoparticles (FeNPs) under proton irradiation, with both in vitro and in vivo models demonstrating nanoparticle-associated enhancement of the biological response [74]. Higher nanoparticle concentrations were associated with reduced cell survival following irradiation with a 45 MeV spread-out Bragg peak (SOBP) proton beam, while in vivo experiments demonstrated greater tumor regression when nanoparticles were combined with proton irradiation than with proton irradiation alone. Although PIXE-related processes were proposed as a mechanism underlying these observations, the biological enhancement cannot be attributed exclusively to PIXE, as additional physicochemical and radiochemical mechanisms may also contribute [74].

The biological response to nanoparticle-assisted proton irradiation may additionally depend on cellular factors. In particular, cell-cycle distribution can influence radiation sensitivity, and treatment-induced alterations in cell-cycle progression may therefore contribute to the observed biological response [76].

#### 3.3.1. Nanoparticles for ROS Upregulation in the Tumor Microenvironment

The tumor microenvironment (TME), the surrounding milieu of a tumor, plays a vital role in tumor proliferation and dissemination. An increasing body of evidence indicates that oxygen deficiency may play a role in the emergence of radioresistance in cancer cells and is considered a primary factor contributing to radiation treatment failure [77]. Methodically engineered nanoparticles could facilitate the production of reactive oxygen species (ROS), leading to increased cytotoxicity and genotoxicity of radiation compared to the radiation-only group. This radiation-sensitizing phenomenon results in enduring DNA double-strand breaks (DSBs), significantly compromising genomic integrity, transitioning from the G2/M phase to the G1/S cell cycle checkpoint, ultimately inducing cellular senescence [78].

Studies indicate that cerium oxide nanoparticles (CONPs) could significantly enhance the vulnerability of pancreatic cancer cells to radiation treatment [79]. CONPs pretreatment selectively enhanced the generation of radiation-induced reactive oxygen species in the acidic acellular milieu and human pancreatic carcinoma cells. In acidic environments, CONPs eliminated superoxide free radicals, although they did not affect the accumulation of hydrogen peroxide. They were shown to eliminate both in a neutral pH setting. The administration of CONP prior to irradiation significantly enhanced the susceptibility of pancreatic cancer cells to apoptosis both in vitro and in vivo, hence challenging the notion that pancreatic tumor growth is independent of normal tissues or host organisms. The hydrogenated nanodiamonds (H-NDs) were evaluated on three human radioresistant cancer cell lines and demonstrated lethal efficacy when subjected to simultaneous radiation. The occurrence of this event is responsible for the induction of DNA damage and the production of reactive oxygen species. The condition appeared to exhibit decreased cell impedance, activation of the G1/S and G2 cell cycle checkpoints, and minimal cell mortality during the initial time period [78].

Consequently, nanoparticles that may elicit substantial ROS production may be utilized as radiosensitizers. We also examined the apoptosis induced by reactive oxygen species resulting from the simultaneous application of nanoparticles and irradiation. Intracellular levels of reactive oxygen species (ROS) were quantified using the specific probe dihydroethidium (DHE). The research indicated that within thirty minutes post-treatment, the intracellular ROS levels in MCF-7 cells subjected to a single application of FA@SeNPs or seed radiation exhibited a minor elevation, thereafter declining to 113.4% and 108% after two hours. Remarkably, the level of ROS surged to 210.2% after thirty minutes and 167.4% during two hours of cultivation with the integration of FA@Se nanoparticles and seed radiation [80]. This type of radiosensitization was likewise conducted on additional intelligent nanoparticles as illustrated in Table 2. Although studies summarized in Table 2 employed mostly non-proton modalities, they are included as mechanistic evidence for nanoparticle-mediated ROS amplification, and therefore they should not be interpreted as direct proof of radiosensitization under proton irradiation.

#### 3.3.2. Nanoparticles for Enhanced Radiation Dose Deposition

The employment of nanoparticles with high atomic number (Z) in radiotherapy is receiving increasing attention due to their remarkable properties in terms of increased radiation dose deposition, but only in the low-energy spectrum, as studies have shown [70,71]. The special features of high-Z nanoparticles, such as their high photoelectric cross section in photon irradiation (Z^4^/E^3^, where Z is the atomic number and E is the incoming photon energy), can result in targeted dose escalation, potentially reducing the radiation dose delivered to normal tissues [71]. Several studies have been increasingly focusing on high-Z nanoparticles used as radiosensitizers such as gold (Au), hafnium (Hf), bismuth (Bi), gadolinium (Gd) and silver (Ag) nanoparticles [6,82].

The increase in the microscopic absorbed dose is small for a change in the macroscopic absorbed dose, as a consequence of the irradiation of the nanoparticle by photon beams, which gives rise to large short-range electrons and Auger cascades [25]. The dose enhancement factor (DEF), which is the scientific expression of the ratio between the absorbed dose in the presence of some specified materials and the absorbed dose in the absence of those materials [83], defines the radiation dose enhancement. For photon irradiation, theoretical analyses have suggested that materials with Z values larger than 70 will give higher dose enhancement. The use of nanoparticles with intermediate Z values (approximately 40–65) may lead to the lowering of DEF for brachytherapy sources or may preserve stability for external kilovolt sources [84].

Photon-based studies have shown that the extent of radiation dosage escalation will be different depending on the dimensions, classifications, nanoparticle concentration, radiation types and energy of the incident beam, among other things. The research suggested that the increase in dosage was more significant for lower photon energies. Furthermore, the highest increment in dose was observed in formulations containing Au, Gd, and I, followed by Fe_2_O_3_ [85]. Also, the dose intensification process in the cancer tissues reduced the effect of photons, thereby reducing the dose on the opposite side of the tumor. The correlation between the nanoparticles’ diameter and DEF was also comparable. For materials with larger dose enhancement effects, the nanoparticle size proved more important in determining the dose enhancement factor. Furthermore, it has been shown that the use of larger amounts of dose-enhancing agents was associated with progressively higher DEF values [85].

Chattopadhyay et al. showed that clonogenic viability of cells treated with human epidermal growth factor receptor-2 (HER-2) targeted gold nanoparticles (Au–T) in combination with X-rays was much lower than cells treated only with X-radiation. This confirms the value of 1.6 in the DEF dimension [86]. In the HT1080 fibrosarcoma cell line, Laurence et al. reported a substantial radioenhancement when combining hafnium oxide nanoparticles and radiation. The clonogenic survival% was significantly decreased using the cobalt-60 source and 6-MV linear accelerator. The dose enhancement factors (DEFs) were found to be 1.4 ± 0.06 and 1.8 ± 0.09 for the 4 Gy and 6-MV accelerator, respectively [87] (Table 3). Similar to Table 2, studies summarized in Table 3 focus on dose-enhancement effects in the context of photon irradiation. Extrapolation of the results to proton therapy necessitates attention, as the underlying radiation-matter interactions may differ.

### 3.4. Challenges and Opportunities in Nanoparticle-Based Radiosensitizers

The extent of nanoparticle accumulation within tumors in vivo is influenced by both passive and active targeting mechanisms and can vary considerably between tumors [88,89]. This is mostly through passive targeting and active targeting. The detection of ligands in combination with tumor-marker chemicals in smart nanoparticles is based on active targeting [88,89]. The EPR effect relies mostly on passive targeting of cancer cells by nanoparticle-mediated drug delivery systems and therefore limits the application of this technology to improve nano-targeted therapy [89]. There is increasing evidence of considerable variability in EPR-mediated tumor targeting, which may be related to differences in tumor vascular permeability and angiogenic activity [89,90]. The observed diversity in tumor accumulation may be partly explained by the generally assumed variability in angiogenic activity associated with EPR activity and cancer severity.

The EPR effect is an important principle in cancer therapy, but the variety of human tumors is a major barrier for the successful clinical application of macromolecular treatments and delivery systems. Furthermore, animal tumors can be generated in a matter of weeks to establish a model for use, while human tumors require a much longer time to mature and show distinct intratumoral heterogeneity of genetic and physiological changes, which are often not apparent in a rapidly growing animal model [89]. To surmount these constraints, innovative methods are required, and the efficiency and impact of drug administration must be improved for cancer treatment. Nanoparticles have a propensity to accumulate in the primary organs of the body, particularly in the liver and kidneys [91]. Determining the appropriate dosage poses a significant challenge in radiation therapy. Elevated doses annihilate cancerous cells, although they concurrently harm the surrounding healthy cells. Consequently, additional research is required to create a safe and efficient nanoparticle-based radiosensitizer for effective clinical use.

### 3.5. Prospective Approaches

Comprehensive and detailed studies are needed to understand the effect of nanoparticles on radiosensitization in proton therapy. The fast proliferation of cancer cells results in tumor sites usually being in a hypoxic microenvironment, which greatly decreases the efficacy of radiation, because the formation of reactive species, such as singlet oxygen, requires the availability of oxygen [77]. Chai et al. developed a radiosensitization system of photosynthetic cyanobacteria capable of in situ oxygen synthesis and nanoscale two-dimensional bismuth to enhance the efficacy of X-ray therapy. The cyanobacteria might change the hypoxic condition of the tumor microenvironment with combined irradiation at 660 nm and X-ray by oxygen production through photosynthesis. Meanwhile, the two-dimensional bismuth radiosensitizer efficiently improved ROS formation and resulted in a significant decrease in cancer proliferation in vivo [92]. The revolutionary treatment approach of modulating the tumor microenvironment with the use of radiosensitizers to augment radiobiological effects opens up avenues for future application in proton therapy to augment its therapeutic efficacy.

Nanoparticles can accumulate in malignant tissues through the enhanced permeability and retention (EPR) effect, although considerable accumulation in healthy tissues may also occur [89]. Surface modification of nanoparticles can facilitate cancer-cell targeting and lead to a more specific biological distribution [88,89]. For example, nanoparticles can be modified with polyethylene glycol (PEG) to reduce their recognition by the immune system and alter their surface properties and distribution in biological systems. Furthermore, interactions between ligands attached to the nanoparticle surface and receptors expressed on cancer-cell membranes can enable more selective targeting [88,89]. Such active-targeting approaches may improve the potential of nanoparticles as radiosensitizers, including their potential application in proton therapy [88,89].

The combination use of radiation therapy with additional therapies also gives significant hope for therapeutic advances. For instance, Fathy et al. described the preparation of chitosan-capped gold nanoparticles (CS-GNPs-dox) loaded with the chemotherapeutic medication Doxorubicin, followed by chemo-radiotherapy enhanced by gold nanoparticle sensitization [93].

Biological investigations have shown that these functionalized gold nanoparticles increase the treatment efficacy against cancers by inducing more DNA double-strand breaks and inducing cell necrosis. The combined irradiation and chemotherapy approach has the advantage of improving the precise application of chemotherapeutic chemicals to malignant cells, as well as increasing the radiosensitizing effect of radiation therapy [93].

## 4. Discussion

Proton treatment has emerged as one of the most sophisticated forms of radiation due to its enhanced dose distribution and capacity to protect adjacent healthy tissues through the Bragg peak effect. In clinical practice, a generic RBE value of approximately 1.1 is conventionally applied; however, proton RBE is not constant and may vary according to LET, dose and biological endpoint [5]. The principal clinical advantage of proton therapy therefore lies in its favorable dose distribution rather than an intrinsically high RBE. In this context, nanoparticles have garnered significant interest as a potential strategy to further enhance the physical and biological effects of proton irradiation [5,6].

Research presented above repeatedly demonstrates that proton radiosensitization by nanoparticles is a complex process including physical, chemical, and biological elements. Proton therapy provides advantages over traditional photon therapy as the dose amplification in photon therapy mainly results from photoelectric interactions, while proton therapy employs additional mechanisms such as the augmented stopping power of protons, improved linear energy transfer (LET), secondary electron production, and localized radical formation around nanoparticles. These routes lead to heightened DNA damage while preserving the intrinsic dosimetric benefits of proton therapy. Among the several nanoparticle formulations investigated, gold nanoparticles are the most extensively researched radiosensitizers. They serve as the standard material for proton radiosensitization, owing to their elevated atomic number, excellent biocompatibility, ease of surface functionalization, and advantageous pharmacokinetic properties [24,25,26,27,28,29,30,31,32,33,34,35,36,37].

An important distinction should be made between evidence obtained specifically under proton irradiation and results derived from conventional photon radiotherapy. This distinction is particularly relevant when considering clinical translation. Hafnium oxide nanoparticles, particularly NBTXR3, represent one of the most clinically advanced nanoparticle radiosensitizers and have undergone clinical evaluation in combination with conventional external-beam radiotherapy [53,54,55]. However, the established clinical evidence has predominantly been obtained with photon-based irradiation and should therefore not be interpreted as direct clinical evidence for nanoparticle-assisted proton therapy [53,54,55]. Published clinical evidence specifically demonstrating nanoparticle-mediated enhancement of proton therapy remains extremely limited, and the current proton-specific evidence is predominantly preclinical [6]. Nevertheless, throughout this review, photon-based studies are included when they provide valuable mechanistic or supportive information regarding nanoparticle-mediated ROS generation, DNA damage, and radiosensitization. However, extrapolation of these findings to proton radiotherapy requires caution, as the underlying radiation–matter interactions differ, and conclusions based exclusively on photon radiotherapy may therefore be misleading [6,51].

It is important to differentiate nanoparticle-induced physical dose enhancement, changes in LET, and changes in biological RBE. Local dose enhancement refers to increased energy deposition in the immediate vicinity of a nanoparticle, commonly quantified using metrics such as DEF or DER, and does not by itself imply an alteration in RBE [51]. Similarly, nanoparticle-induced changes in proton energy loss may modify the local LET or generate secondary electrons with highly localized energy deposition [25,51]. Although LET is an important determinant of proton RBE, an increase in LET cannot be directly interpreted as an increase in RBE, since RBE additionally depends on dose, biological endpoint, cell or tissue type, and reference radiation [5]. Most nanoparticle–proton studies reviewed here demonstrate nanoscale physical dose enhancement, increased ROS production, or enhanced DNA damage and cell killing, rather than directly measuring RBE against a reference radiation [26,29,43,51]. Therefore, the currently available evidence primarily supports nanoparticle-mediated enhancement of local physical and biological effects, while definitive demonstration and quantification of nanoparticle-induced changes in proton RBE remain limited.

The interpretation of the available evidence should take into account the experimental level at which nanoparticle-mediated enhancement has been demonstrated. Monte Carlo simulations provide valuable information on nanoscale energy deposition and secondary-particle production but cannot establish a biological radiosensitizing effect [25,42,51,68]. Similarly, DNA- or molecular-level experiments demonstrate radiation-induced damage under controlled conditions but do not necessarily predict cellular or tumor responses [43]. In vitro studies provide direct evidence of cellular radiosensitization [26,29], whereas in vivo models additionally incorporate nanoparticle biodistribution, tumor accumulation, clearance, and normal-tissue effects [27]. Consequently, nanoscale physical dose enhancement should not be interpreted as equivalent to an increase in macroscopic tumor dose or improved tumor control [51]. Evidence of the latter requires validation in appropriate biological and ultimately clinical models.

The amalgamation of gold nanoparticles with proton irradiation has demonstrated a significant reduction in tumor cell viability, enhanced production of reactive oxygen species, elevated occurrences of DNA double-strand breaks, and augmented suppression of tumor proliferation in vivo [26,27,28,29,30,31,32,33,34,35,36,37]. Furthermore, Monte Carlo simulations have reliably forecasted the nanoscale dose increase near gold nanoparticles; nonetheless, the extent of macroscopic dose enhancement remains rather modest [25,28,32,33].

Various metallic nanoparticles, including platinum, gadolinium, silver, bismuth, and iron, have demonstrated promising radiosensitizing properties. Platinum nanoparticles hold significant potential due to their inherent chemotherapeutic properties and substantial physical dose amplification. Gadolinium nanoparticles possess the additional advantage of serving as MRI contrast agents, enhancing theranostic applications [44,45,46,47,49,50]. Similarly, bismuth nanoparticles have demonstrated exceptionally high levels of ROS production and enhanced radiosensitization, indicating that additional high-Z substances might eventually rival or perhaps surpass gold nanoparticles in focused therapeutic uses [29].

Metal oxide nanoparticles represent a burgeoning and significant category of proton radiosensitizers. Hafnium oxide nanoparticles have progressed into multiple clinical trials, including photon irradiation, and demonstrate advantageous biocompatibility and stability. Despite the scarcity of biological evidence regarding protons, early studies indicate significant potential. Nanoparticles of titanium dioxide, iron oxide, tantalum oxide, and cerium oxide have demonstrated their capacity to enhance ROS production, induce DNA damage, and facilitate localized dose delivery during proton irradiation [51,61,68]. Significantly, numerous materials include diagnostic imaging functionalities, hence presenting opportunities for image-guided proton therapy. In addition to the enhancement of the physical dosage, a particularly attractive aspect of nanoparticle-assisted proton therapy is the modulation of the tumor microenvironment, as highlighted in the literature [77].

Nanoparticles that may generate oxygen or modulate reactive oxygen species (ROS), including cerium oxide, selenium, manganese dioxide, and hydrogenated nanodiamonds, have demonstrated potential to enhance radiation response through mechanisms involving oxidative stress and associated biological damage [78,79,80,81]. These findings indicate that biological radiosensitization could contribute to the overall therapeutic advantage, alongside physical dose enhancement [78,79,80,81].

The available evidence does not indicate that atomic number alone determines the suitability of a nanoparticle for proton radiosensitization. This distinction is particularly important when extrapolating findings from photon radiotherapy. Under kilovoltage X-ray irradiation, high-Z materials benefit strongly from the Z-dependent photoelectric interaction, whereas proton interactions are governed predominantly by charged-particle energy-loss processes and show a substantially weaker dependence on atomic number [6,13,14,15]. Among the nanoparticles investigated specifically with proton irradiation, gold nanoparticles currently have the most extensive evidence base, encompassing Monte Carlo simulations, in vitro studies, and in vivo experiments, together with favorable properties for synthesis and surface functionalization [26,27,28,29,30,31,32,33,34,35,36,37]. Platinum nanoparticles are also particularly promising and have produced greater local dose enhancement than gold in some comparative simulations, although their biological evidence remains less extensive [42,43]. Gadolinium nanoparticles show reproducible radiosensitizing effects, but generally lower physical enhancement than gold or platinum [45,46,47,48]. Importantly, metal oxide nanoparticles such as titanium dioxide demonstrate that efficient proton radiosensitization may also arise through enhanced ROS production and surface-catalytic effects rather than high atomic number alone [51]. Therefore, the most promising nanoparticles for proton therapy should not be selected solely according to Z, but according to a combination of physical dose enhancement, radiochemical activity, biological response, tumor targeting, biocompatibility, and achievable intratumoral concentration [6].

Despite encouraging preclinical findings, the clinical translation of nanoparticle-assisted proton therapy remains limited for several interconnected reasons. First, nanoparticle accumulation observed in experimental tumor models may not accurately reflect that achieved in patients. The EPR effect is highly heterogeneous in human tumors and depends on tumor vascularisation, stromal characteristics, interstitial pressure, and tumor type, making nanoparticle delivery less predictable than in rapidly growing experimental models [89,90]. Moreover, nanoparticles may accumulate in non-target organs, particularly the liver, spleen, and kidneys, raising concerns regarding long-term toxicity and clearance [91]. A further limitation is the considerable heterogeneity among published studies with respect to nanoparticle composition, size, concentration, surface functionalization, proton energy, cellular models, and biological endpoints, which complicates comparison between studies and the establishment of reproducible dose–response relationships. Among these variables, nanoparticle concentration and intracellular distribution appear to be particularly important determinants of radiosensitization, since they directly influence the amount and spatial distribution of material available for interaction with the proton beam [85]. Particle size and aggregation state further affect cellular uptake, intracellular localization, and the spatial distribution of secondary electrons and reactive species [85,94,95]. Proton energy and the position of the nanoparticles relative to the Bragg peak are also important physical determinants, as proton energy loss and LET vary along the beam path. Surface functionalization may indirectly influence radiosensitization by modifying nanoparticle stability, cellular uptake, tumor targeting, and intracellular localization [15,85]. Consequently, differences in nanoparticle concentration, size, localization, and irradiation conditions may account for a substantial proportion of the variability among published radiosensitization results, although the relative contribution of each parameter cannot yet be reliably ranked because systematic head-to-head comparisons remain limited. Finally, most available evidence derives from Monte Carlo simulations and in vitro experiments, whereas robust in vivo studies and clinical data remain scarce. Together with challenges related to long-term biocompatibility, large-scale reproducible manufacturing, and regulatory requirements, which also represent broader translational challenges for nanomaterial-based biomedical technologies, these factors help explain why the promising radiosensitizing effects observed under controlled preclinical conditions have not yet resulted in widespread clinical implementation [96,97].

Future research should therefore follow a stepwise translational approach. Nanoparticle design should prioritize predictable clearance, biocompatibility, and minimal long-term toxicity, while optimizing surface functionalization to achieve reproducible and selective tumor accumulation. Importantly, radiosensitization should be demonstrated at nanoparticle concentrations that are realistically achievable in vivo rather than only under optimized in vitro conditions. Proton-specific experimental protocols should also be standardized with respect to nanoparticle characterization, concentration, proton energy, LET, position within the spread-out Bragg peak, dosimetry, and biological endpoints to improve reproducibility and comparison across studies. Promising formulations should subsequently be validated in appropriate in vivo tumor models, with systematic assessment of pharmacokinetics, biodistribution, clearance, normal-tissue toxicity, intratumoral nanoparticle concentration, and therapeutic efficacy before progression to early-phase clinical evaluation [89,96].

Beyond these translational priorities, the integration of proton therapy with oxygen-generating nanoparticles, immunomodulatory agents, chemotherapy, or targeted molecular therapies could potentially improve therapeutic results while maintaining acceptable safety standards. Concurrently, standardized experimental methodologies and therapeutically pertinent animal models will be essential for the conversion of promising laboratory findings into conventional clinical practice.

## 5. Conclusions

Nanoparticles present a compelling approach to enhance the physical and biological impacts of proton treatment, hence augmenting its therapeutic effectiveness. Recent studies indicate that certain nanoparticle systems, particularly high-Z metallic and metal oxide nanoparticles, can enhance localized dose delivery, stimulate the production of reactive oxygen species, increase DNA damage, and surmount tumor radioresistance. Nanoparticles with multifunctional capabilities that can transport medications to specific targets, modulate gene expression, or react to radiation offer additional opportunities to enhance treatment selectivity and reduce systemic toxicity. Preclinical findings are encouraging; nevertheless, the transition to clinical use is obstructed by challenges related to biodistribution, tumor targeting, long-term safety, and standardization. The efficacy and safety of nanoparticle-assisted proton therapy for precise cancer treatment must be substantiated by future interdisciplinary research and meticulously structured clinical trials.

## Figures and Tables

**Table 1:** Comparative summary of representative proton-specific studies of nanoparticle-mediated radiosensitization, according to experimental level and irradiation conditions.

Study/Nanoparticle	NP Characteristics/Concentration	Evidence Level/Model	Proton Irradiation	Main Endpoint	Main Finding	Main Limitation	Ref.
Polf et al.—AuNPs	AuNP diameter 44 ± 8 nm; targeted intracellular delivery	In vitro; DU145 prostate cancer cells	Clinical proton irradiation	Clonogenic survival; RBE	Proton effectiveness increased by approximately 15–20% in AuNP-containing cells	Single cell line; specialized targeting system; in vitro RBE does not establish tumor-level benefit	[26]
Kim et al.—metallic NPs (Au/Fe)	100–300 mg/kg intravenously	In vivo mouse tumor model + in vitro ROS assessment	Proton irradiation, single doses 10–41 Gy; Bragg peak either within or beyond tumor	Complete tumor regression; survival; ROS	CTR increased by 37–62% or 50–100%, depending on irradiation configuration; 1-year survival improved versus proton alone	High NP exposure; animal model; mechanism not attributable solely to physical dose enhancement	[27]
Rashid et al.—Au, Pt, SPION, Bi_2_O_3_	Different NP materials and morphologies; 1 mmol/L	In vitro; HCT116 colon cancer cells	150 MeV protons	Clonogenic survival; SER; ROS	SER: Bi_2_O_3_ 4.93, Pt 3.08, Au 2.64, SPION 1.95	Differences in particle size/shape/material prevent attribution of effect to atomic number alone	[29]
Ganjeh & Salehi—Au, Pt, Ag, I, Ta-based NPs	Various materials	Monte Carlo simulation	3–15 MeV protons	Physical dose enhancement	Pt produced the greatest calculated enhancement among the compared materials	Simulation only; biological relevance and clinically achievable NP concentrations uncertain	[42]
Zwiehoff et al.—PtNPs	PtNPs combined with albumin, Tween or PEG stabilizing ligands	Molecular/plasmid DNA experiment	Proton doses of 2 and 5 Gy	DNA double-strand damage	>55% clustered DNA damage obtained with optimized PtNP–ligand combinations	Molecular-level evidence; does not demonstrate cellular or tumor radiosensitization	[43]
Gerken et al.—Au, HfO_2_, TiO_2_ and other NPs	Multiple metallic/metal-oxide NPs	Monte Carlo + in vitro cellular and ROS experiments	100 MeV protons	Physical DEF; ROS; biological radiosensitization	Au physical enhancement fell to near baseline under proton irradiation, while TiO_2_ and HfO_2_ retained biological enhancement related largely to catalytic/ROS effects	In vitro; biological enhancement cannot be explained by physical dose alone	[51]
Brero et al.—magnetic iron-oxide NPs	Magnetic nanoparticles	In vitro; BxPC3 pancreatic cancer cells	Clinical proton beam	Cell survival; DSBs; ROS	Magnetic NPs enhanced proton-induced biological damage, with stronger effects when combined with hyperthermia	Hyperthermia confounds the isolated NP–proton effect; single cell line	[61]
McKinnon et al.—Ta_2_O_5_, CeO_2_, Au	100 nm spherical NPs	Geant4/Geant4-DNA nanoscale simulation	5 and 50 MeV protons	Local energy deposition	14–27% local nanoscale enhancement; ceramic NPs ~15% versus ~25% for Au	Purely nanoscale simulation; does not establish macroscopic dose enhancement or biological benefit	[68]

Abbreviations: Au, gold; AuNPs, gold nanoparticles; Bi_2_O_3_, bismuth oxide; CeO_2_, cerium oxide; DEF, dose enhancement factor; DSB, DNA double-strand break; HfO_2_, hafnium oxide; NP, nanoparticle; PEG, polyethylene glycol; Pt, platinum; PtNPs, platinum nanoparticles; RBE, relative biological effectiveness; ROS, reactive oxygen species; SER, sensitization enhancement ratio; SPION, superparamagnetic iron oxide nanoparticles; Ta_2_O_5_, tantalum pentoxide; TiO_2_, titanium dioxide. Quantitative values are reported as described in the original studies. Where specific experimental parameters were not reported or could not be reliably extracted from the original publication, they were not inferred. Physical nanoscale dose enhancement should not be interpreted as equivalent to macroscopic tumor-dose enhancement or improved biological response.

**Table 2:** NP-based radiosensitizers to upregulate the levels of reactive oxygen species.

Reference	NP-Type	Size (nm)	Radiation (Radiation Dose)	Cell Line
Grall et al. (2015) [78]	Hydrogenated nanodiamonds	16	γ-ray (4 Gy)	Caki-1, ZR75.1Ku70wt,
Wason et al. (2013) [79]	Cerium oxide nanoparticles	5–8	Ionizing radiation (5 Gy)	Human pancreatic cancer cell line L3.6 pl
Yang et al. (2017) [80]	Folic acid-conjugated selenium nanoparticles	192	125I seeds	Human breast cancer MCF-7 cells
Liu et al. (2020) [81]	Manganese dioxide nanoparticles	126.1 ± 9.6	X-ray (5 Gy)	Human non-small cell lung cancer H1299 cells and human head and neck squamous cell carcinoma SCC7 cells

**Table 3:** Different types of high-Z NP-based radiosensitizers involving DEF determination.

Reference	High-Z Element	NP-Type	Size (nm)	Radiation (Radiation Dose)	Cell Line	DEF Value
Chattopadhyay et al. (2013) [86]	Au	HER-2-Au NPs	30	X-ray (11 Gy)	K-BR-3 breast cancer cells	1.6
Maggiorella et al. (2012) [87]	Hf	Hafnium oxide NPs	50	Cobalt-60 (4 Gy)	Human HT1080 fibrosarcoma cell line	1.4 ± 0.06 (4 Gy); 1.8 ± 0.09 (6 MV)

## Data Availability

No new data were created or analyzed in this study. Data sharing is not applicable to this article.

## Abbreviations

The following abbreviations are used in this manuscript:

ASO	Antisense Oligonucleotide
ATM	Ataxia-Telangiectasia Mutated
AuNPs	Gold Nanoparticles
CT	Computed Tomography
DEF	Dose Enhancement Factor
DER	Dose Enhancement Ratio
DNA	Deoxyribonucleic Acid
DSB	Double-Strand Break
EGFR	Epidermal Growth Factor Receptor
EPR	Enhanced Permeability and Retention
Gy	Gray
HER-2	Human Epidermal Growth Factor Receptor 2
High-Z	High Atomic Number
IR	Ionizing Radiation
LET	Linear Energy Transfer
Low-Z	Low Atomic Number
MRI	Magnetic Resonance Imaging
NP	Nanoparticle
NPs	Nanoparticles
PET	Positron Emission Tomography
PIGE	Particle-Induced Gamma-Ray Emission
PIXE	Particle-Induced X-ray Emission
PLGA	Poly(lactic-co-glycolic acid)
PT	Proton Therapy
RBE	Relative Biological Effectiveness
ROS	Reactive Oxygen Species
SOBP	Spread-Out Bragg Peak
SSB	Single-Strand Break
TME	Tumor Microenvironment
